# Comparing biobehavioral profiles across two social stress paradigms in children with and without autism spectrum disorders

**DOI:** 10.1186/2040-2392-3-13

**Published:** 2012-11-17

**Authors:** Blythe A Corbett, Clayton W Schupp, Kimberly E Lanni

**Affiliations:** 1 Department of Psychiatry, Vanderbilt University, Vanderbilt Kennedy Center, PMB 40, 230 Appleton Place, Nashville, TN, 37203, USA; 2 Cancer Prevention Institute of California, 2201 Walnut Avenue, Suite 300, Fremont, CA, 94538, USA; 3 Veterans Affairs Northern California Healthcare System, 10535 Hospital Way, Building 649, Mather, CA, 95655, USA

**Keywords:** Cortisol, Autism, Stress, Novelty, Peer, Age

## Abstract

**Background:**

Autism spectrum disorders (ASD) are defined by impairment in reciprocal social interaction and flexible adaptation to the environment. This study compared physiological stress in children with and without ASD exposed to two social stress protocols. We hypothesized that the ASD group would show heightened initial and enduring cortisol levels to the social stressors, which would be moderated by age and intelligence.

**Methods:**

Twenty-seven children with ASD and 32 with typical development (TYP) completed a standardized social-evaluative performance task and a validated paradigm of social play with peers. Physiological stress was measured by salivary cortisol at nine time points. Statistical approaches included repeated-measures linear mixed models and correlation analyses.

**Results:**

The average cortisol level of both groups during initial exposure to social situations was significantly greater than baseline levels (ASD, *P* = 0.018; TYP, *P* = 0.006). Stress responsivity was significantly different between the groups; the TYP group showed a significant reduction in cortisol over time (*P* = 0.023), whereas the ASD group maintained an elevated cortisol level (*P* >0.05). The ASD group evidenced greater variability in between-group, within-group and intra-individual analyses. Age was a positive moderator of stress for the ASD group (*P* = 0.047), whereas IQ was a negative moderator for the TYP group (*P* = 0.061).

**Conclusions:**

Initial stress to novel social scenarios is idiosyncratic and predictive of subsequent exposure. Amidst significant variability in cortisol, children with ASD show enhanced and sustained social stress that increases with age. Developmental and cognitive factors differentially moderate stress in children with ASD and TYP, respectively. A model of neuroendocrine reactivity is proposed.

## Background

Autism spectrum disorders (ASD) refer to a group of pervasive developmental disorders marked by impairment in social interaction, verbal and nonverbal communication and flexible adaptation to the changing environment [1]. Perhaps as a result of challenges with social communication and social perception, many children with ASD experience anxiety [2] and physiological arousal in social situations [3,4]. Moreover, elevated and variable arousal and stress responsivity may be an important moderator in symptom profile [5,6]. The aim of the current study is to evaluate cortisol, one of several biological stress responses, in children with ASD and in children with typical development (TYP) to determine how social engagement, intellectual functioning, and age contribute to inter-individual and intra-individual variability in children with ASD across different social contexts.

When an individual experiences increased arousal in response to a perceived stressor, the limbic–hypothalamic–pituitary–adrenal (LHPA) axis is engaged. Activation of limbic structures, such as the amygdala, hippocampus and prefrontal cortex (PFC), set into motion a neuroendocrine cascade controlled by neurons in the hypothalamic paraventricular nucleus. When the system is activated, the regulatory peptides arginine vasopressin and corticotropin-releasing hormone are released into the pituitary portal, increasing circulation of adrenocorticotrophic hormone, which drives glucocorticoid action for the release of cortisol in humans (for example [7,8]).

Importantly, this system is elegantly adaptive such that following a stress response glucocorticoid action is inhibited, allowing the system to return to a homeostatic state. Furthermore, stress is often thought of as idiosyncratic because a number of factors may contribute to whether or not an event is deemed stressful to the individual, including novelty, age, gender, socioeconomic status, and context. It is within this framework that the following investigation exploring variability in the biological stress response in ASD is presented and interpreted.

Children with ASD are driven by a persistent need for sameness and have difficulty adapting to change [9]. Studies examining cortisol as a biomarker of stress show enhanced arousal in children with ASD, suggesting hyperresponsivity to acute, novel stress rather than a pattern of chronic hyperarrousal (for example [4,10-13]). Significant variability in the diurnal rhythm (inter-individual and intra-individual differences) and responsivity (individual differences in responding to environmental conditions) of cortisol has also been documented [5,10], suggesting subgroups of stress responders within the autism spectrum in relation to daily stressors and sensory functioning (for example [6]).

Since ASD are defined by impairment in social communication, exploring social stress is critical. Heightened cortisol levels have been reported in response to school integration [14] and social unfamiliarity [4]. We recently investigated physiological stress associated with social play (see peer interaction described below) [3]. This paradigm emulates a natural playground setting to ascertain whether this benign situation is biologically stressful. Indeed, many children with ASD responded with significantly higher levels of cortisol [3,15], and distinct biobehavioral profiles emerged in children who exhibited a significant stress response compared with home baseline levels and typically developing children based on developmental (older children), biological (elevated cortisol responder), and behavioral (social interaction) patterns. Importantly, the enhanced cortisol response was observed in children who willingly engaged in interaction; the enhanced cortisol level therefore does not support the notion of a response to social threat.

In contrast, a standardized laboratory-based psychosocial stress test, the Trier Social Stress Test – child version (TSST-C) [16] – known to activate the LHPA axis in typically developing children (for example [17,18]) and in children with various medical conditions (for example [16,19]) – does not significantly increase cortisol in many children with ASDs [11,20,21]. We replicated this finding [22], while also noting a wide range of cortisol variability in children with ASD. Cumulatively, the attenuated stress response in these studies suggests that the aspect of this protocol that elicits LHPA activation in TYP children may not be perceived by children with ASD, reflecting differences in perception and stress responsivity in ASD or perhaps suggesting a blunted response secondary to chronic stress. As with many biobehavioral aspects of ASD, there exists a wide range of variability in the stress response of children with ASD, suggesting moderating factors [3].

Taken together, the findings result in a double dissociation such that the LHPA axis is perturbed in children with ASD by unique, seemingly benign, social and nonsocial stressors yet they do not exhibit a neuroendocrine response to a standardized social-evaluative stressor. These paradoxical findings warrant closer investigation, which may be carried out by exposing the same children to both social paradigms.

The current study explored neuroendocrine responsivity in children with and without ASD exposed to two stress protocols, to comprehensively investigate novelty (cortisol levels during initial exposure to stress), stress responsivity (cortisol response across the different stressors), variability (between-group, within-group and intra-individual differences), moderators (such as age and IQ), and associations (linear correlations of stress responsivity between and within the groups).

## Methods

### Participants

The combined study included 59 nonmedicated, male children between 8 and 12 years of age, which included 27 ASD children (22 autism, 5 pervasive developmental disorder – not otherwise specified) and 32 TYP children. Demographic information is presented in Table 1. The participants were part of one or both previous studies [3,22]. Some participants that completed one protocol were not willing or able to participate in the other; therefore, only children that completed both protocols were included in the final correlational analyses (14 ASD children, 8 TYP children). Diagnosis was based on all of the following: the Diagnostic and Statistical Manual criteria [1]; a previous diagnosis by an experienced psychologist, psychiatrist, or behavioral pediatrician; clinical judgment (of BAC); and corroborated by a total score on the Autism Diagnostic Observation Scale [23] at or above the ASD threshold for Module 3. 

**Table 1:** Demographic variables

	**Age**				**IQ**			
	**Mean**	**SD**	**Range**	***t*score**	**Mean**	**SD**	**Range**	***t*score**
Autism	10.1	1.3	8.0 to 12.6	*t*(57) = −0.40^NS^	94.9	18.1	75 to 128	*t*(55) = −7.01***
								
Neurotypical	9.9	1.6	8.1 to 12.5		122.6	11.4	99 to 142	

NS, not significant; SD, standard deviation. ***P <0.0005.

Prepubescent participants were enrolled, defined as a cumulative score ≤5 in each of three categories – voice, pubic hair, and facial hair – on the Pubertal Development Scale [24]. All participants had estimated IQ ≥75 [25].

Approval for the current study was obtained via the Institutional Review Board at the University of California, Davis, and in compliance with the Code of the Ethical Principles for Medical Research Involving Human Subjects of the World Medical Association (Declaration of Helsinki). Fully informed written consent from parents and verbal assent from participants were obtained.

### Procedures

The study included two social paradigms: a standardized psychosocial performance task of social evaluative threat [22]; and a validated peer interaction paradigm of social play with peers [3]. The order of the protocols was randomized across participants, and occurred on separate days at least 1 month apart.

#### Peer Interaction Playground Paradigm

**Figure 1:**
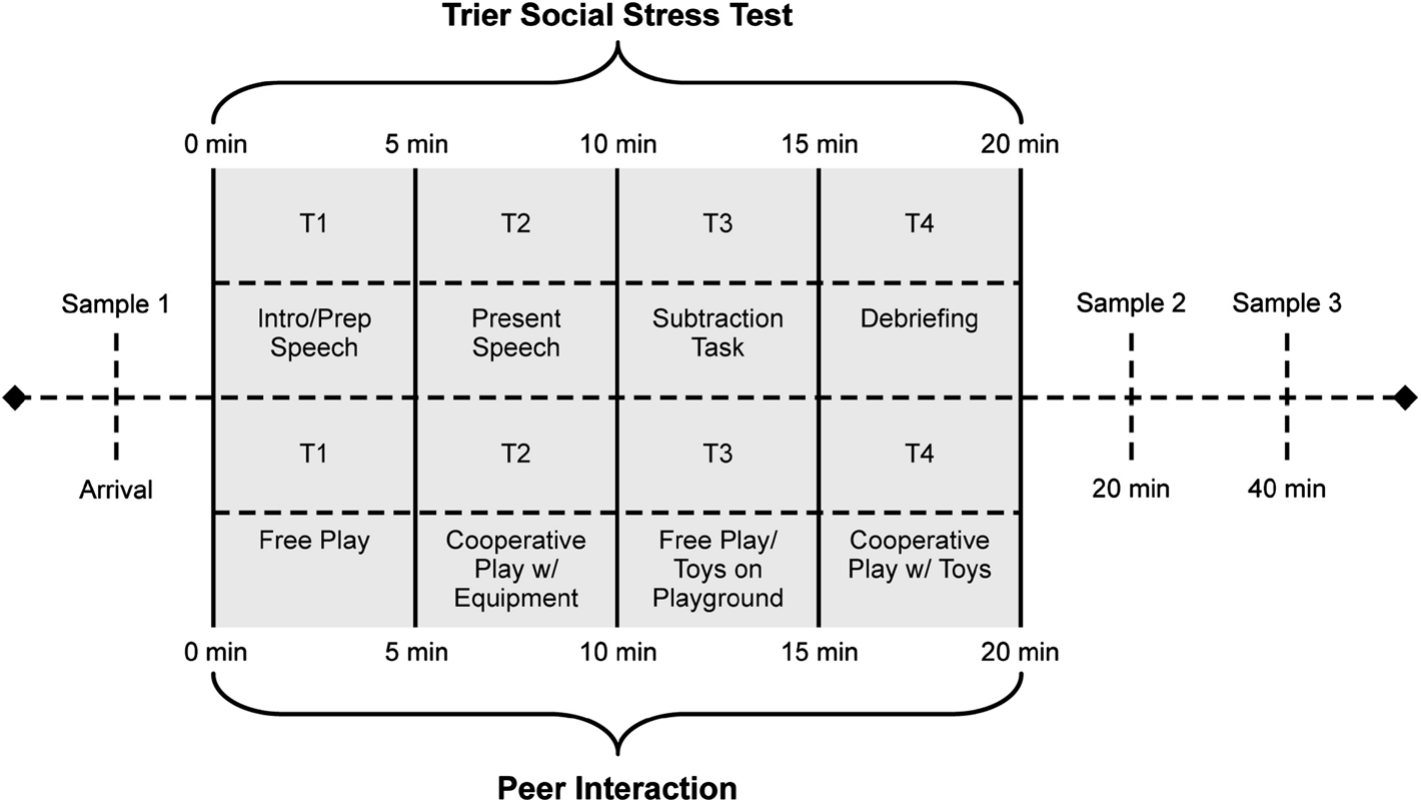
**Experimental timeline and cortisol sampling.** Cortisol sampling occurred at three time points during each 20-minute stress protocol. Sample 1 occurred immediately prior to beginning the 20-minute stressor, Sample 2 occurred upon completion of the stressor, and Sample 3 was taken 20 minutes following completion of the stressor. This figure also identifies the tasks completed within each 5-minute period of the stress protocols (that is, T1 = minutes 0 to 5; T2 = minutes 6 to 10; T3 = minutes 11 to 15; T4 = minutes 16 to 20), with the Trier Social Stress Test – child version (TSST-C) displayed above and the Peer Interaction Playground Paradigm (PEER) displayed below the timeline.

The Peer Interaction Playground Paradigm (PEER) is an ecologically valid playground protocol that allows detailed behavioral observation in a non-intrusive, natural setting (see [3]). The paradigm includes a child with ASD, a TYP child, and an age-matched and gender-matched confederate. The 20-minute protocol blends intermittent 5-minute periods of free and cooperative play, allowing considerable flexibility for natural behavior to occur (see Figure 1). 

The confederate provides behavioral structure to the free play, permitting key interactive sequences to occur. As shown in Figure 1, the 20-minute play session was divided into four, 5-minute time periods: T1 = free play, T2 = confederate solicits interaction on the play structures, T3 = free play, and T4 = confederate solicits interaction with toys. The confederate wore earphones, enabling direct communication with research personnel who provided directive cues instructing him when to approach the participants (T2 and T4) and when to engage in independent play (T1 and T3).

#### Trier Social Stress Test – child version

The TSST-C is a standardized psychosocial stress protocol that is also comprised of four 5-minute time periods (20 minutes in total): T1 = introduction and speech preparation, T2 = speech delivery, T3 = serial subtraction, and T4 = debriefing (see Figure 1) [16]. Two committee members who remain affectively neutral throughout the procedure instruct the participant to prepare and perform a short-story speech task and spontaneously complete a serial subtraction task that will be evaluated in comparison with his peers. Following completion the committee debriefs the participant, warmly congratulating him and stating that his performance was not truly judged.

### Cortisol sampling and assay procedures

Salivary cortisol is a valid and reliable measure of diurnal rhythm and physiological arousal [26]. Physiological stress can be measured in salivary cortisol with consideration of a lag in detectable responsivity of approximately 20-minutes. Samples were collected at 20- and 40-minutes post stressor to reflect response to initial (novelty) and continuing exposure (stress) to the social stressors, respectively, for comparison in the current study (Figure 1). Samples were also collected at additional time points to evaluate the diurnal regulation of cortisol from the home and to use the average daily afternoon samples to obtain a comprehensive baseline for each child. With the exception of the afternoon values, these samples are not included in the analyses for the current study; however, the interested reader is directed to these prior studies [3,22].

Our standardized passive drool collection procedures were followed, which are also detailed elsewhere [3,5]. Briefly, parents were trained for the collection of home samples in person and via video instruction. Research personnel obtained all laboratory samples. For collection, participants were provided with Trident® Original gum, (Cadbury Adams, Parsippany, NJ, USA) to act as a salivary stimulant and then they deposited 1 ml saliva through a straw into a test tube. A pre-prepared label was subsequently attached to the test tube and the precise time of collection documented on it. Samples were stored in a −20°C freezer, thawed and centrifuged at 6,000 rpm for 10 minutes. Assays were performed using coated-tube radioimmunoassay kits (Siemens Medical Solutions Diagnostics, Los Angeles, CA, USA) as previously described [3]. Samples were assayed in a single large batch run.

#### Average afternoon cortisol

As noted above, research participants completed home sampling at four time points (that is, waking, 30 minutes post waking, afternoon and evening) for six diurnal cycles (24 samples), with initial results presented elsewhere [5]. Importantly, average afternoon cortisol was calculated based on the six afternoon values for each participant taken during the study to represent the participant’s typical afternoon cortisol level for comparison with stress protocols. The arrival values might not represent an accurate baseline measurement for some participants due to a variety of extraneous factors (for example, anticipatory stress); therefore, the 20-minute and 40-minute values were adjusted for this measurement in the models.

### Statistical analyses

The current study explored neuroendocrine responsivity in children with and without ASD exposed to two stress protocols, to comprehensively investigate novelty (cortisol levels during initial exposure to stress), stress responsivity (cortisol response across the different stressors), variability (between-group, within-group and intra-individual differences), moderators (such as age and IQ), and associations (linear correlations of stress responsivity between and within the groups).

Between-group analyses were performed across all demographic, diagnostic, and inclusion variables using independent two-sample *t* tests if the assumption of normality held true; otherwise, the equivalent nonparametric test was used. Variances were thus compared using Levene’s test of homogeneity to determine whether the equal variance assumption between the groups was valid. If the equal variance assumption was not met, the Welch–Satterthwaite degrees of freedom (df) approximation was used.

To investigate novelty, stress responsivity and variability, the 20-minute and 40-minute post-stressor cortisol values, respectively, were adjusted for average home afternoon levels, and then analyzed using linear mixed-effects random intercepts models. The random effects models allowed comparison of cortisol variability. Error terms between subjects were assumed to be independent, normally distributed, and to have common variance within each group (autism vs. the typically developing) while the variances between the groups were allowed to vary. The model with unequal group variances was compared with a reduced model assuming equal variances using a likelihood ratio test. Initial models explored all two-way and three-way interactions between diagnosis, sample time, and type of stressor. To evaluate potential moderators, age and IQ were centered on the within-group average levels and included as potential effect modifiers of diagnosis. Additional two-way interactions between diagnostic group and age and IQ were included in the final model, which allowed the modifying effect to differ between groups.

Finally, to examine associations across the variables, Pearson correlations were calculated for subjects that participated in both paradigms. Each subject had three cortisol measurements per study (arrival, 20- minutes, 40- minutes) and the relative time point was compared between the studies (for example, PEER arrival compared with TSST-C arrival).

## Results

We explored neuroendocrine responsivity in ASD children and TYP children exposed to two social stress protocols, to more thoroughly investigate novelty, stress responsivity, variability, moderators and associations. Participants that completed either study were included to broadly examine social stress in and across groups to determine between-group, within-group, and intra-individual differences. Additional analyses included a subset of participants that completed both protocols to directly compare and contrast stress responsivity with each protocol. Salivary cortisol measurements are positive and skewed toward large values; a log transformation was therefore performed to achieve approximate normality, and log-cortisol values were used in all analyses.

### Variability

Cortisol responses were evaluated across the social stress protocols by analyzing the 20-minute and 40-minute values while adjusting for average afternoon cortisol by taking all cortisol values for each subject and subtracting that individual’s average afternoon cortisol from the diurnal study. This approach allows each child’s stress value to be compared with his own expected baseline levels, where significant values greater than zero could signify a stress response. The PEER and TSST-C studies were combined and analyzed using a mixed-effect random intercepts model, allowing the groups to have different variances.

The intra-class correlation for the ASD group was 58.9% while for the TYP group it was 69.3%, suggesting that very large portions of total variance is explained by differences between the children. In addition, the ASD group had a between-child variance that was 1.63 times greater than the TYP group and a within-child variance that was 2.54 times greater than the TYP group, suggesting significantly greater heterogeneity in the ASD group (*P* = 0.0009). In summary, there was significant variability between the groups (ASD vs. TYP), within the ASD group, as well as intra-individual differences in the children with ASD.

### Novelty

**Figure 2:**
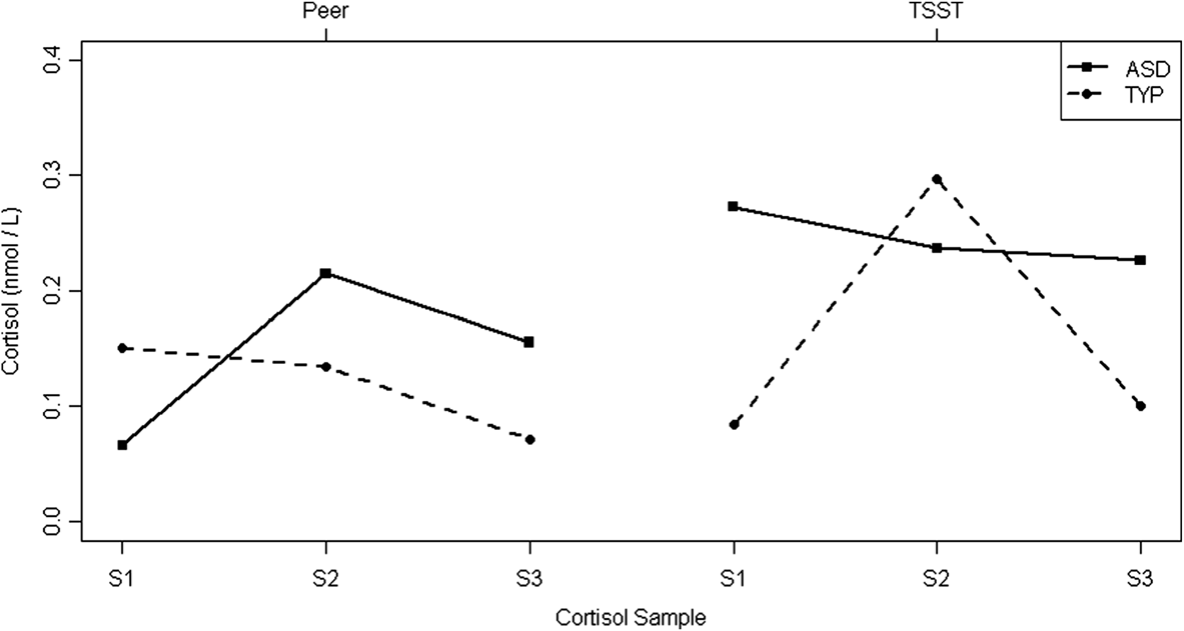
**Cortisol responsivity to the TSST**-**C and PEER during initial exposure to the stressor.** Cortisol levels (minus expected afternoon levels) during initial exposure to the Peer Interaction Playground Paradigm (PEER; *x* axis) and cortisol levels (minus expected afternoon levels) during the Trier Social Stress Test – child version (TSST-C; *y axis*). Black circles, children with autism spectrum disorders (ASD). Both groups showed strong correlations between the 20-minute cortisol values across the social stressors. TYP, typical development.

Both groups exhibited average cortisol levels at the beginning of the stressors that were significantly greater than the average afternoon levels (ASD, β = 0.22, *t* = 2.42, *P* = 0.018; TYP, β = 0.24, *t* = 2.88, *P* = 0.006), although between-group comparison of this response was not significantly different (*P* >0.05). These values represent the average group level means at the 20-minute time point, adjusted for individual average afternoon levels. As displayed in Figure 2, this effect seemed particularly prominent for the ASD group on the afternoon of the TSST-C, perhaps reflecting the elusive nature of this task (that is, the participant arrived expecting to complete a challenging academic task) relative to the PEER (that is, the participant arrived expecting to play on a playground).

### Stress

Although members of each group showed higher cortisol levels at initial exposure, the groups had significantly different slopes to those at the 40-minute time point (see Figure 2). Specifically, the cortisol levels of the TYP group quickly reduced, demonstrating robust recovery following initial arousal and indicating that the actual stressor did not perturb them. In contrast, the ASD group maintained an elevated cortisol level as indicated by a very shallow slope from 20-to-40 minutes that was not significantly different from zero, whereas the TYP group had a significant decrease in cortisol (β = −0.12, *t* = −2.31, *P* = 0.023). There was a 51% drop in cortisol for the TYP group while the ASD group experienced a 17% decrease.

### Moderators

Based on between-group comparisons, age was an important moderator for the ASD group, with older children exhibiting higher than average levels of cortisol (β = 0.15, *t* = 2.03, *P* = 0.047); however, age was not found to be a significant moderator for the TYP group. The β value represents the additional log-cortisol, on average, for each additional year of age for a child in the ASD group, equivalent to a 75% increase in average cortisol above expected afternoon levels.

Evaluation of cognitive level as a potential moderator of physiological responsivity showed that IQ was not significant for the ASD group; however, it was a marginally significant moderator for the TYP group, such that children with higher IQ experienced slightly lower cortisol levels (β = −0.014, *t* = −1.91, *P* = 0.061) to initial exposure. The β value represents the decrease in log-cortisol for each additional point in IQ, such that for a 10-point increase in IQ the average 20-minute value was 56% lower than for a TYP child with average IQ levels.

Additional exploration revealed that for a TYP child of average IQ, there was no appreciable difference in initial response (at 20-minutes) to either the PEER or the TSST-C; however, as IQ increased, the child had a lower initial response to the PEER (β = −0.020, *t* = −2.81, *P* = 0.007) and a higher response to the TSST-C (β = 0.026, *t* = 3.33, *P* = 0.001). The response to the stressor (at 40-minutes) remained consistently lower regardless of the task and was not affected by IQ. To put this in perspective, a TYP child that had an IQ 10 points above average would have an initial response to the PEER at a level 90% lower than the TYP child of average IQ, and an initial response to the TSST-C 30% higher than the TYP child of average IQ. They would then, regardless of protocol or IQ, experience a roughly 55% drop in cortisol in response to the stressor (slope from 20-to-40 minutes). Despite the starting value at 20 minutes based on IQ and type of stressor, the slope from 20 to 40 minutes remained equal because nothing moderated the 40-minute value.

### Associations

**Figure 3:**
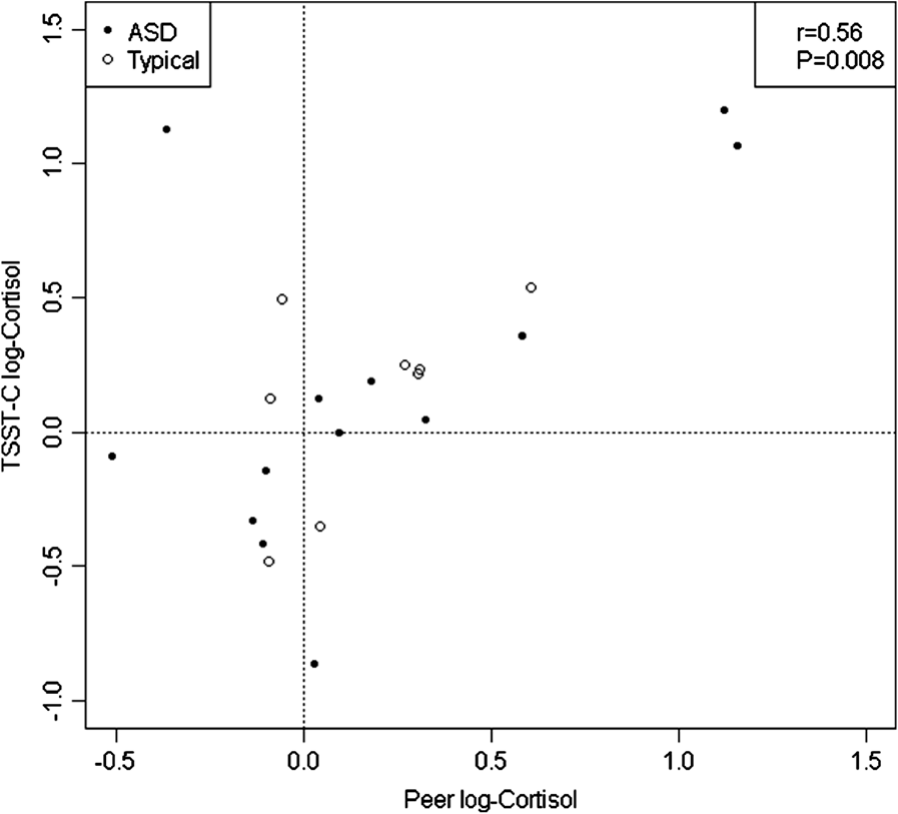
**Cortisol response profiles.** Mean log-cortisol response profiles by diagnosis and stressor type after adjustment for individual average afternoon levels.

Correlational analyses revealed that cortisol arrival levels between the groups were moderately correlated across experiments (*P* = 0.027, *r* = 0.47, df = 20), ostensibly driven by anticipatory stress in children with ASD (*P* = 0.032, *r* = 0.57, df = 12) compared with the TYP group (*P* = 0.43, *r* = 0.28, df = 6). The 20-minute post value in each experiment was strongly correlated (*P* = 0.008, *r* = 0.56, df = 19; see Figure 3) during the early exposure to stressors, thus reflecting heightened response to novelty in the children with ASD (*P* = 0.044, *r* = 0.56, df = 11) versus the TYP group (*P* = 0.16, *r* = 0.55, df = 6). However, the 40-minute post value was not correlated across conditions, between the groups (*P* = 0.91, *r* = 0.03, df = 20) or within the groups (*P* >0.05), suggesting a differential response based on the conditions of the stressor and between the groups.

## Discussion

The purpose of the study was to thoroughly characterize the physiological response to social stress as measured by salivary cortisol in children with ASD compared with same-age peers to increase our understanding of factors that contribute to individual variability. The comprehensive analyses revealed substantial between-group, within-group, and intra-individual variability in ASD. While both groups showed an increase in cortisol upon novel exposure to the stressors, the ASD group demonstrated an elevated and prolonged stress response. Age was a strong, positive moderator of stress for children with ASD, whereas IQ differentially moderated stress response for the TYP group.

One of the more consistent findings in ASD is heterogeneity in many areas of biological, behavioral and neural functioning (for example [6,27-30]). We observed significant differences in variability in cortisol responses between the groups such that children with ASD showed a greater range of cortisol values than typically developing peers. Additionally, much of the total variance was explained by large within-group and intra-individual differences in ASD despite enrolling a homogeneous, well-characterized group.

The initial or novel responses to the paradigms were highly correlated, indicating that children in both groups with a heightened response to one paradigm similarly showed heightened cortisol to the other stressor. While this finding appears strongly driven by the ASD group, the initial response does not seem specific to ASD but may be more of an individual trait. In other words, initial cortisol responsivity to novel social scenarios in children with ASD appears idiosyncratic and may be driven by other factors suggesting more of a trait phenomenon. Additionally, the novelty response was similar regardless of the type of social stressor. Although there were some outliers, children who responded with a higher or lower cortisol response to the TSST-C showed a comparable response with the other paradigm. Further, since the order was randomized and carried out in different settings, order effects or habituation to research personnel cannot explain the findings.

Children with ASD often show atypical patterns of sensitivity to stimuli (for example [31]). The current findings also demonstrate responsivity to novelty that transcends the specific social paradigm. This implies that response to novelty is inherently associated with basal LHPA axis functioning, resulting in a direct biological effect regardless of exposure to a given stressor or psychological factors that influence stress responsivity. Recent studies in healthy adults indicate that temperamental avoidance of novelty is associated with heightened cortisol, whereas novelty-seeking is inversely associated with cortisol levels [32]. These effects are distinct from psychologically mediated factors, which may occur in response to a specific psychosocial stressor, such as the TSST-C. Corticotropin-releasing hormone may play a pivotal role in this association because central administration results in neophobia as well as reduced social behavior and exploration in various animal species [33,34].

The predilection of many children with ASD to avoid novel situations may be analogous to a temperamental characteristic and considered on a developmental continuum. Physiological arousal is closely linked with stable, temperamental characteristics. For example, children, adults, and nonhuman primates shown to be behaviorally inhibited – such that they withdraw from novel situations – evidence LHPA axis hyperactivity [35,36].

Although children in both groups showed stress responsivity to novelty, the children with ASD exhibited a persistent stress response whereas the TYP group exhibited a more rapid recovery, suggesting less perturbation by the stressor itself. The more rapid decline in cortisol in the TYP group could reflect robust negative feedback following initial evaluation. Alternatively it could reflect a more modest primary activation relative to the group baseline. In contrast, many children with ASD showed enhanced and sustained physiological arousal to the social stressors. As noted above, there remains variability in the stress response that is clearly dependent on the interpretation of the event as being threatening. If the event is fundamentally not perceived as threatening, the response will be diminished. In short, not all social events are equally stressful for individuals with ASD. Furthermore, it is acknowledged that the actual response to the stressor is also influenced by the initial interpretation and response to the novel situation. As such, while we aim to distinguish the novelty and stress responses, they are closely linked and further influenced by context and other modifying factors (for example [37]).

Since attention, perception and interpretation of stimuli are necessary to elicit a stress response, investigating factors that may moderate physiological arousal is a valuable although seldom used approach. The current study demonstrated that age is a very strong moderator in ASD such that for each additional year of age there was a 75% increase in cortisol level above the expected afternoon baseline levels. This association was not observed in neurotypical peers and supports prior literature that with increasing age comes concomitant insight into social challenges, contributing to higher rates of anxiety and stress in youth with ASD [2-4,38,39]. Although cortisol levels tend to increase with development and puberty [40], participants were prepubescent, and this relationship was not observed in the TYP group. Age as a potent moderator of physiological arousal in older children with ASD therefore certainly warrants additional study.

We also evaluated whether general cognitive functioning might contribute to the perception of and response to social stressors. Results showed that IQ was a significant moderator for the TYP group, such that children with higher IQ experienced lower cortisol responsivity to novelty. Moderation by IQ during initial exposure was not observed in the ASD group, in TYP children of average intellect nor found at the latter cortisol response to the stressor. Intriguingly, when evaluating the paradigms separately, cortisol levels in the higher IQ TYP children were differentially expressed based on the social paradigm such that they had a lower initial response when given the chance to play with peers yet a higher initial response when facing social evaluation. Since perception of the respondent is crucial to trigger a stress response, it is meaningful that children with higher cognitive functioning perceived the peer interaction as nonthreatening whereas social judgment was threatening. Because the IQ for the TYP group ranged from 99 to 142, such an increase in relation to the lowered cortisol implies it may be biologically meaningful and not simply a spurious finding. If replicated, this relationship suggests that cognitive appraisal is an important moderator of stress, particularly for those with higher intellect.

**Figure 4:**
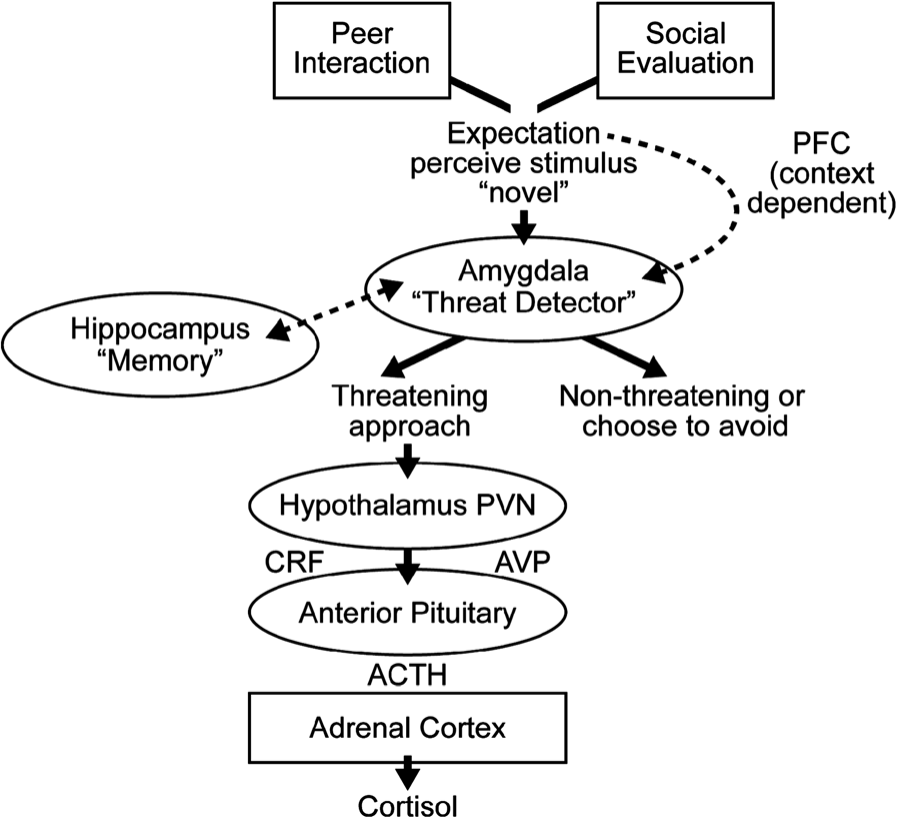
**Neuroendocrine cascade.** Representation of the neuroendocrine cascade including factors proposed to influence a stress response derived from the current findings. The two social paradigms, peer interaction and social evaluation, are the potential stressors. The amygdala, an early threat detector, is highly responsive to novel stimuli, which is engaged in social and affective appraisal. If the individual perceives the event to be stressful, the amygdala may become engaged, initiating a physiological response. However, the prefrontal cortex (PFC), which plays a pivotal role in cognitive appraisal, can influence reactivity by information gleaned from the context in which the stressor occurs. The individual may perceive the event as nonthreatening or choose to avoid it, thereby aborting a heightened response to the stressor. Based on developmental factors including personal experiences, the hippocampus may also become engaged. Limbic structures are thus influential in triggering the activation of the hypothalamic–pituitary–adrenal (HPA) axis for the cascade of neuropeptides and eventual release of cortisol from the adrenal cortex.

In consideration of current and previous findings, a model of neuroendocrine responsivity related to social behavior in ASD is proposed (see Figure 4). The figure represents the neuroendocrine cascade including factors proposed to influence a stress response. In this model, the two social paradigms – peer interaction and social evaluation – are the potential stressors. It is well established that when an individual experiences a heightened level of arousal in response to a perceived stressor, activation of limbic structures such as the amygdala, hippocampus and PFC sets into motion a neuroendocrine cascade [7]. The amygdala is an early threat detector that is highly responsive to novel stimuli and engaged during social and affective appraisal. If the individual perceives the event to be stressful, the amygdala may become engaged initiating a physiological response. Importantly, the PFC, which plays a pivotal role in cognitive appraisal, can influence reactivity by information gleaned from the context in which the stressor occurs. An individual may perceive the event as nonthreatening or choose to avoid it, thereby aborting a heightened response to the stressor. Based on developmental factors including personal experiences, the hippocampus may engage triggering the activation of the limbic-hypothalamic–pituitary–adrenal axis for the cascade of neuropeptides and eventual release of cortisol from the adrenal cortex. 

Several brain structures involved in the neuroendocrine cascade have been implicated in the neuropathology of ASD and may influence the biobehavioral patterns observed. The amygdala, a primary activator of stress responsivity for novel processive stimuli [7], has been hypothesized to contribute to increased anxiety and stress in individuals with autism [41,42]. This temporal lobe structure is instrumental in social and affective regulation, and it is differentially activated in ASD (for example [43-45]). As a rapid, initial-response threat detector, the amygdala plays a pivotal role during early exposure to stressors and may contribute to enhanced cortisol levels in response to novel events.

The PFC, an important neural structure involved in social and cognitive appraisal of a stressor [46,47], may also be implicated in autism (for example [48,49]). Since the PFC is vital for cognitive control, insight, and context-dependent reasoning, it may contribute to the differential stress responses of the neurotypical children with higher IQ. Additionally, heightened activation initially triggered by the amygdala may not be inhibited by the PFC due to a dysfunctional circuit or reduced connectivity between the amygdala and PFC in ASD [50], leading to a prolonged stress response in dynamic social contexts in ASD.

Finally, volumetric differences in the hippocampus, a structure involved in memory formation and retrieval, have been reported (for example [51-53]). As such, developmental and personal experiences may enhance stress responsivity in older children with ASD. The model displayed is a visual representation of the findings and theoretical notions proposed herein; the model is not intended to summarize the complexity of the LHPA axis and other factors that impinge on an organism’s response to potential stressors.

To summarize, the current investigation aimed to more thoroughly investigate different aspects of cortisol responsivity in children with ASD. Limitations include a somewhat modest sample size, a rather restricted age range due to developmental and hormonal factors, and inclusion of only male participants (owing to higher male-to-females ratios (4:1) in ASD and hormonal differences based on gender). In addition, the mean IQ of the comparison group reflected higher than average intellectual ability. While the current study utilized salivary cortisol to evaluate the LHPA axis, other indices such as salivary α-amylase may also be useful indicators of sympathetic adrenomedullary activity in response to stress (for example [54]). It may be worthwhile for future studies to evaluate both the LHPA axis and sympathetic adrenomedullary system in conjunction to expand our understanding of the stress response in children with ASD, which have been examined in other studies [55]. Additionally, further exploration into the association between perceived emotional stress or anxiety and the physiological response to stress might assist in addressing concerns regarding how to best measure anxiety in children with ASD (for example [56]) and have important implications for treatment. In consideration of these limitations, the generalizability of the findings must be constrained. Efforts are underway to expand our research protocols based on age and gender while including other indices of stress responsivity.

## Conclusions

This comprehensive investigation of social stress serves to better characterize the biobehavioral stress profiles in ASD and TYP children. The notion that higher IQ in typically developing children may moderate stress has important implications for research to consider how perception and cognitive functioning influence stress responsivity. For both groups, response to novelty is idiosyncratic but a good predictor for reactivity to new situations, thereby emphasizing the importance of preparing highly responsive children for upcoming events with schedules or other preparatory coping strategies. In ASD, the significant variability in cortisol confirms the heterogeneity in this population and the need to individualize interventions that strongly consider stress reactivity in the child’s treatment program. This observation is punctuated by the fact that even mild social situations can result in heightened and prolonged stress responsivity that intensify with development and experience. Exploration into better characterizing cortisol levels in children with ASD has shown the importance of considering initial and enduring responses to potential social stressors that may contribute to how children with ASD adapt to the dynamic social world.

## Abbreviations

ASD: autism spectrum disorders; df: degrees of freedom; LHPA: limbic–hypothalamic–pituitary–adrenal; PEER: Peer Interaction Playground Paradigm; PFC: prefrontal cortex; TSST-C: Trier Social Stress Test – child version; TYP: typical development.

## Competing interests

The authors declare that they have no competing interests.

## Authors’ contributions

BAC conceptualized the investigation, engaged in study design, supervised participant enrollment, provided data interpretation and completed the initial draft of the manuscript. CWS participated in study design, completed all statistical analyses, and participated in data interpretation and preparation of the manuscript. KEL conducted comprehensive assessments of research participants, engaged in study development and participated in the writing of the manuscript. All authors contributed to and approved the final manuscript.
